# Understanding Unreported Cases in the COVID-19 Epidemic Outbreak in Wuhan, China, and the Importance of Major Public Health Interventions

**DOI:** 10.3390/biology9030050

**Published:** 2020-03-08

**Authors:** Zhihua Liu, Pierre Magal, Ousmane Seydi, Glenn Webb

**Affiliations:** 1 School of Mathematical Sciences, Beijing Normal University, Beijing 100875, China; zhihualiu@bnu.edu.cn; 2 Université de Bordeaux, IMB, UMR 5251, F-33400 Talence, France; 3 CNRS, IMB, UMR 5251, F-33400 Talence, France; 4 Département Tronc Commun, École Polytechnique de Thiés, Thies 21001, Senegal; oseydi@ept.sn; 5 Mathematics Department, Vanderbilt University, Nashville, TN 37212, USA; glenn.f.webb@vanderbilt.edu

**Keywords:** corona virus, reported and unreported cases, isolation, quarantine, public closings, epidemic mathematical model

## Abstract

We develop a mathematical model to provide epidemic predictions for the COVID-19 epidemic in Wuhan, China. We use reported case data up to 31 January 2020 from the Chinese Center for Disease Control and Prevention and the Wuhan Municipal Health Commission to parameterize the model. From the parameterized model, we identify the number of unreported cases. We then use the model to project the epidemic forward with varying levels of public health interventions. The model predictions emphasize the importance of major public health interventions in controlling COVID-19 epidemics.

## 1. Introduction

An epidemic outbreak of a new human coronavirus, termed the novel coronavirus COVID-19, has occurred in Wuhan, China. The first cases occurred in early December, 2019, and, by 29 January 2020, more than 7000 cases had been reported in China [1]. Early reports advise that COVID-19 transmission may occur from an infectious individual, who is not yet symptomatic [2]. Evidently, such asymptomatic infectious cases are not reported to medical authorities. For epidemic influenza outbreaks, reported cases are typically only a fraction of the total number of the symptomatic infectious individuals. For the current epidemic in Wuhan, it is likely that intensive efforts by Chinese public health authorities have reduced the number of unreported cases.

Our objective is to develop a mathematical model, which recovers from data of reported cases, the number of unreported cases for the COVID-19 epidemic in Wuhan. For this epidemic, a modeling approach has been developed in [3], which did not consider unreported cases. Our work continues the investigation in [4,5] of the fundamental problem of parameter identification in mathematical epidemic models. We address the following fundamental issues concerning this epidemic: How will the epidemic evolve in Wuhan with respect to the number of reported cases and unreported cases? How will the number of unreported cases influence the severity of the epidemic? How will public health measures, such as isolation, quarantine, and public closings, mitigate the final size of the epidemic?

## 2. Results

### 2.1. The Model and Data

Our model consists of the following system of ordinary differential equations:

(1) $$\left\{\begin{array}{c} S^{\prime }\left(t\right)=-\tau S\left(t\right)\left[I\left(t\right)+U\left(t\right)\right],\\ I^{\prime }\left(t\right)=\tau S\left(t\right)\left[I\left(t\right)+U\left(t\right)\right]-\nu I\left(t\right),\\ R^{\prime }\left(t\right)=\nu_{1}I\left(t\right)-\eta R\left(t\right),\\ U^{\prime }\left(t\right)=\nu_{2}I\left(t\right)-\eta U\left(t\right). \end{array}\right.$$

Here, $t\geq t_{0}$ is time in days, $t_{0}$ is the beginning date of the epidemic, S(t) is the number of individuals susceptible to infection at time *t*, I(t) is the number of asymptomatic infectious individuals at time *t*, R(t) is the number of reported symptomatic infectious individuals (i.e., symptomatic infectious with sever symptoms) at time *t*, and U(t) is the number of unreported symptomatic infectious individuals (i.e., symptomatic infectious with mild symptoms) at time *t*. This system is supplemented by initial data

(2) $$S\left(t_{0}\right)=S_{0}>0,\;I\left(t_{0}\right)=I_{0}>0,\;R\left(t_{0}\right)=0\;\mathrm{and}\;U\left(t_{0}\right)=U_{0}\geq 0.$$

**Figure 1:**
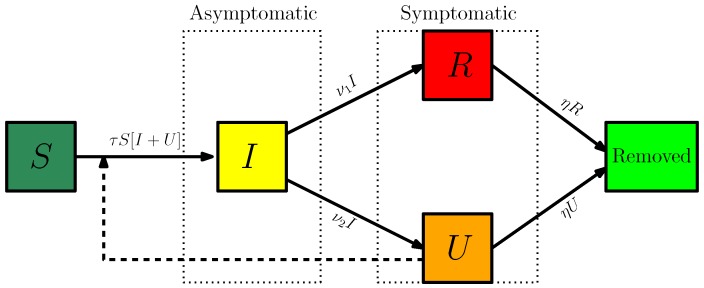
Diagram flux.

Figure 1 is the flow chart of the model (1). The parameters of the model are listed in Table 1.

We use three sets of reported data to model the epidemic in Wuhan: First, data from the Chinese CDC for mainland China (Table 2), second, data from the Wuhan Municipal Health Commission for Hubei Province (Table 3), and third, data from the Wuhan Municipal Health Commission for Wuhan Municipality (Table 4). These data vary but represent the epidemic transmission in Wuhan, from which almost all the cases originated in the larger regions.

### 2.2. Comparison of Model (1) with the Data

For influenza disease outbreaks, the parameters τ, ν, $\nu_{1}$, $\nu_{2}$, η, as well as the initial conditions $S(t_{0})$, $I(t_{0})$, and $U(t_{0})$, are usually unknown. Our goal is to identify them from specific time data of reported symptomatic infectious cases. To identify the unreported asymptomatic infectious cases, we assume that the cumulative reported symptomatic infectious cases at time *t* consist of a constant fraction along time of the total number of symptomatic infectious cases up to time *t*. In other words, we assume that the removal rate ν takes the following form: $\nu =\nu_{1}+\nu_{2}$, where $\nu_{1}$ is the removal rate of reported symptomatic infectious individuals, and $\nu_{2}$ is the removal rate of unreported symptomatic infectious individuals due to all other causes, such as mild symptom, or other reasons.

The cumulative number of reported symptomatic infectious cases at time *t*, denoted by CR(t), is

(3) $$CR\left(t\right)=\nu_{1}\int_{t_{0}}^{t}I\left(s\right)ds.$$

Our method is the following: We assume that CR(t) has the following special form:

(4) $$CR\left(t\right)=\chi_{1}\exp\left(\chi_{2}t\right)-\chi_{3}.$$

We evaluate $\chi_{1},\chi_{2},\chi_{3}$ using the reported case data in Table 2, Table 3 and Table 4. We obtain the model starting time of the epidemic $t_{0}$ from (4):

$$CR\left(t_{0}\right)=0\Leftrightarrow \chi_{1}exp\left(\chi_{2}t_{0}\right)-\chi_{3}=0\;\Rightarrow \;t_{0}=\frac{1}{\chi_{2}}\left(\ln\left(\chi_{3}\right)-\ln\left(\chi_{1}\right)\right).$$

We fix $S_{0}=11.081\times 10^{6}$, which corresponds to the total population of Wuhan. We assume that the variation in S(t) is small during the period considered, and we fix ν,η,f. By using the method in Section 4, we can estimate the parameters $\nu_{1},\nu_{2},\tau$ and the initial conditions $U_{0}$ and $I_{0}$ from the cumulative reported cases CR(t) given (4). We then construct numerical simulations and compare them with data.

**Figure 2:**
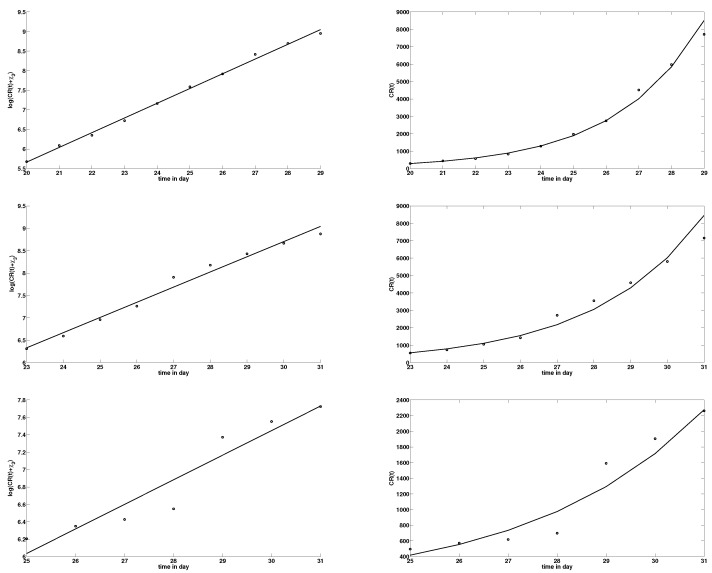
In the left side figures, the dots correspond to $t\to \ln\left(CR\left(t\right)+\chi_{3}\right)$, and in the right side figures, the dots correspond to t→CR(t), where CR(t) is taken from the cumulated confirmed cases in Table 2 (**top**), in Table 3 (**middle**), and in Table 4 (**bottom**). The straight line in the left side figures corresponds to $t\to \ln\left(\chi_{1}\right)+\chi_{2}t$. We first estimate the value of $\chi_{3}$ and then use a least square method to evaluate $\chi_{1}$ and $\chi_{2}$. We observe that the data for China in Table 2 and Hubei in Table 3 provides a good fit for CR(t) in (4), while the data for Wuhan in Table 4 does not provide a good fit for CR(t) in (4).

The evaluation of $\chi_{1}$, $\chi_{2}$, $\chi_{3}$, and $t_{0}$, using the cumulative reported symptomatic infectious cases in Table 2, Table 3 and Table 4, is shown in Table 5 and in Figure 2 below.

**Remark** **1.** *According to Table 2, Table 3 and Table 4, the time t=0 will correspond to 31 December. Thus, in Table 5, the value $t_{0}=5.12$ means that the starting time of the epidemic is 5 January, the value $t_{0}=-2.45$ means that the starting time of the epidemic is 28 December, and $t_{0}=-4.52$ means that the starting time of the epidemic is 26 December.*

**Remark** **2.** *As long as the number of reported cases follows (1), we can predict the future values of CR(t). For $\chi_{1}=0.16$, $\chi_{2}=0.38$, and $\chi_{3}=1.1$, we obtain*

$$\begin{array}{cccccccc} 30\;\mathrm{January} & 31\;\mathrm{January} & 1\mathrm{February} & 2\;\mathrm{February} & 3\;\mathrm{February} & 4\;\mathrm{February} & 5\;\mathrm{February} & 6\;\mathrm{February}\\ 8510 & 12,390 & 18,050 & 26,290 & 38,290 & 55,770 & 81,240 & 118,320 \end{array}$$

*The actual number of reported cases for China are 8163 confirmed for 30 January, 11,791 confirmed for 30 January, and 14,380 confirmed for 1 February. Thus, the exponential formula (4) overestimates the number reported after day 30.*

From now on, we fix the fraction *f* of symptomatic infectious cases that are reported. We assume that between 80% and 100% of infectious cases are reported. Thus, *f* varies between 0.8 and 1. We assume 1/ν, and the average time during which the patients are asymptomatic infectious varies between one day and seven days. We assume that 1/η, the average time during which a patient is symptomatic infectious, varies between one day and seven days. Thus, we fix f,ν,η. Since *f* and ν are known, we can compute

(5) $$\nu_{1}=f\nu \;\mathrm{and}\;\nu_{2}=\left(1-f\right)\nu .$$

Moreover, by following the approach described in the supplementary information, we should have

(6) $$I_{0}=\frac{\chi_{1}\chi_{2}exp\left(\chi_{2}t_{0}\right)}{f\;\nu }=\frac{\chi_{3}\chi_{2}}{f\;\nu },$$

(7) $$\tau =\frac{\chi_{2}+\nu }{S_{0}}\frac{\eta +\chi_{2}}{\nu_{2}+\eta +\chi_{2}},$$

and

(8) $$U_{0}=\frac{\nu_{2}}{\eta +\chi_{2}}I_{0}=\frac{(1-f)\nu }{\eta +\chi_{2}}I_{0}.$$

By using the approach described in the supplementary material, the basic reproductive number for model (1) is given by

$$R_{0}=\frac{\tau S_{0}}{\nu }\left(1+\frac{\nu_{2}}{\eta }\right).$$

By using $\nu_{2}=\left(1-f\right)\;\nu$ and (7), we obtain

(9) $$R_{0}=\frac{\chi_{2}+\nu }{\nu }\frac{\eta +\chi_{2}}{\left(1-f\right)\;\nu +\eta +\chi_{2}}\left(1+\frac{(1-f)\;\nu }{\eta }\right).$$

### 2.3. Numerical Simulations

We can find multiple values of η, ν and *f* which provide a good fit for the data. For application of our model, η, ν and *f* must vary in a reasonable range. For the corona virus COVID-19 epidemic in Wuhan at its current stage, the values of η, ν and *f* are not known. From preliminary information, we use the values

f=0.8,η=1/7,ν=1/7.

**Figure 3:**
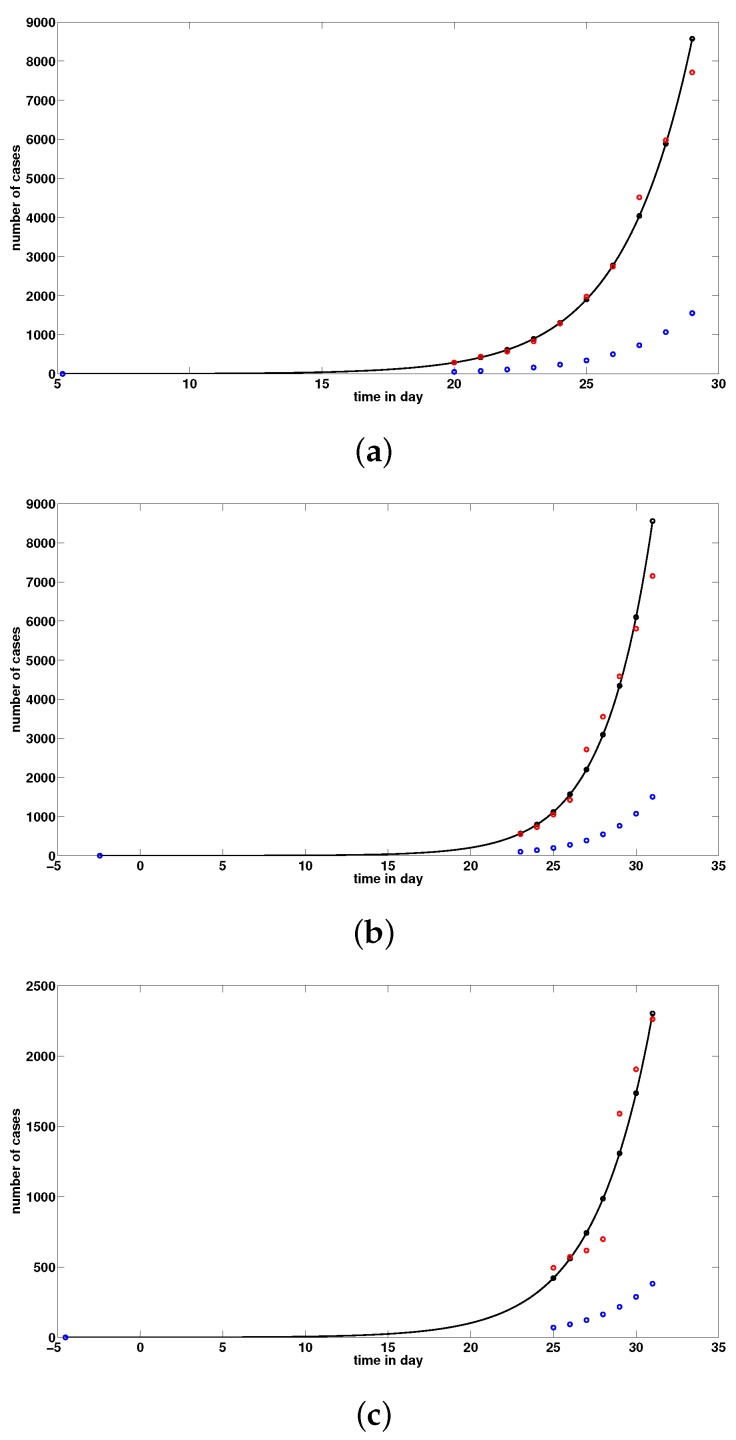
In these figures, we use f=0.8,η=1/7,ν=1/7, and $S_{0}=11.081\times 10^{6}$. The remaining parameters are derived by using (6)–(8). In (**a**), we plot the number of t→CR(t) (black solid line) and t→U(t) (blue dotted) and the data (red dotted) corresponding to the confirmed cumulated cases for mainland China in Table 2. We use $\chi_{1}=0.16$, $\chi_{2}=0.38$, $\chi_{3}=1.1$, $t_{0}=5.12$ and $S_{0}=11.081\times 10^{6}$ which give $\tau =4.44\times 10^{-08}$, $I_{0}=3.62$, $U_{0}=0.2$ and $R_{0}=4.13$. In (**b**), we plot the number of t→CR(t) (black solid line) and t→U(t) (blue dotted) and the data (red dotted) corresponding to the confirmed cumulated case for Hubei province in Table 3. We use $\chi_{1}=0.23$, $\chi_{2}=0.34$, $\chi_{3}=0.1$ and $t_{0}=-2.45$ and $S_{0}=11.081\times 10^{6}$ which give $\tau =4.11\times 10^{-08}$$I_{0}=0.3$, $U_{0}=0.02$ and $R_{0}=3.82$. In (**c**), we plot the number of t→CR(t) (black solid line) and t→U(t) (blue dotted) and the data (red dotted) corresponding to the confirmed cumulated cases for Wuhan in Table 4. We use $\chi_{1}=0.36$, $\chi_{2}=0.28$, $\chi_{3}=0.1$, $t_{0}=-4.52$, and $S_{0}=11.08\times 10^{6}$, which give $\tau =3.6\times 10^{-08}$, $I_{0}=0.25$, $U_{0}=0.02$, and $R_{0}=3.35$.

By using formula (9) for the basic reproduction number, we obtain from the data in Table 2 that $R_{0}=4.13.$ Using model (1) and the values in Table 5, we plot the graph of t→CR(t), t→U(t) and the data for the confirmed cumulated cases in Figure 3, according to Table 2 for China, Table 3 for Hubei, and Table 4 for Wuhan. We observe from these figures that the data for China and Hubei fit the model (1), but the data for Wuhan do not fit the model (1) because the model (4) is not a good model for the data for Wuhan in Table 4. The data for Wuhan do not fit an exponential function.

In what follows, we plot the graphs of t→CR(t), t→U(t), and t→R(t) for Wuhan by using model (1). We define the turning point $t_{p}$ as the time at which the red curve (i.e., the curve of the non-cumulated reported infectious cases) reaches its maximum value. For example, in the figure below, the turning point $t_{p}$ is day 54, which corresponds to 23 February for Wuhan.

In the following, we take into account the fact that very strong isolation measures have been imposed for all China since 23 January. Specifically, since 23 January, families in China are required to stay at home. In order to take into account such a public intervention, we assume that the transmission of COVID-19 from infectious to susceptible individuals stopped after 25 January. Therefore, we consider the following model: for $t\geq t_{0}$,

(10) $$\left\{\begin{array}{c} S^{\prime }\left(t\right)=-\tau \left(t\right)S\left(t\right)\left[I\left(t\right)+U\left(t\right)\right],\\ I^{\prime }\left(t\right)=\tau \left(t\right)S\left(t\right)\left[I\left(t\right)+U\left(t\right)\right]-\nu I\left(t\right)\\ R^{\prime }\left(t\right)=\nu_{1}I\left(t\right)-\eta R\left(t\right)\\ U^{\prime }\left(t\right)=\nu_{2}I\left(t\right)-\eta U\left(t\right) \end{array}\right.$$

where

(11) $$\tau \left(t\right)=\left\{\begin{array}{c} 4.44\times 10^{-08},\;\mathrm{for}\;t\in \left[t_{0},25\right],\\ 0,\;\mathrm{for}\;t>25. \end{array}\right.$$

**Figure 4:**
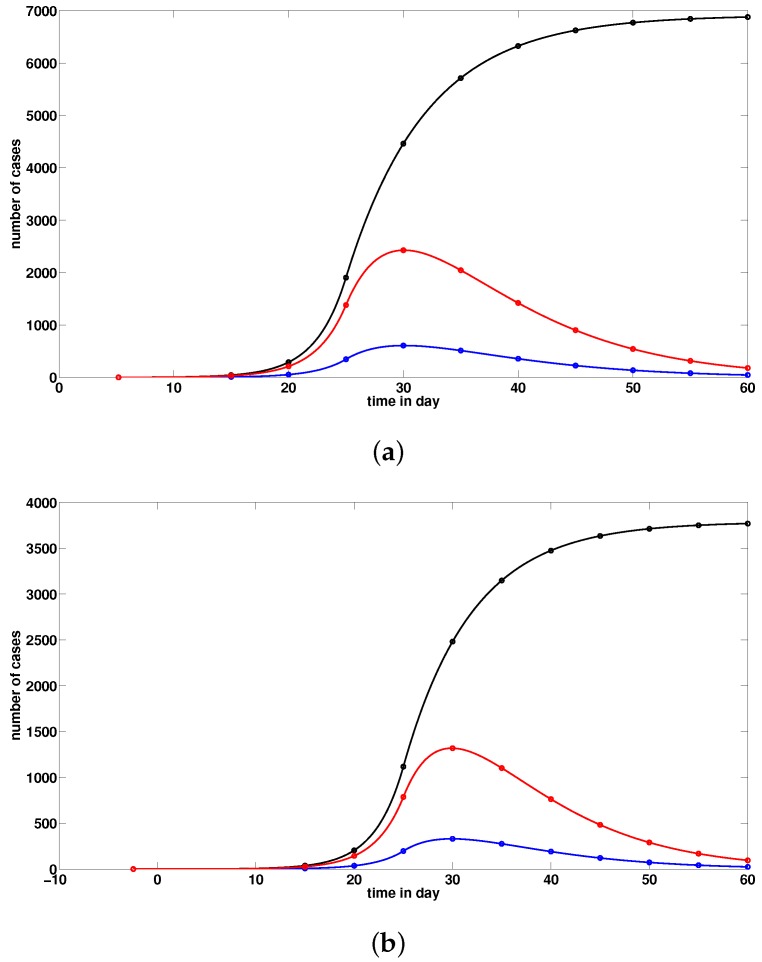

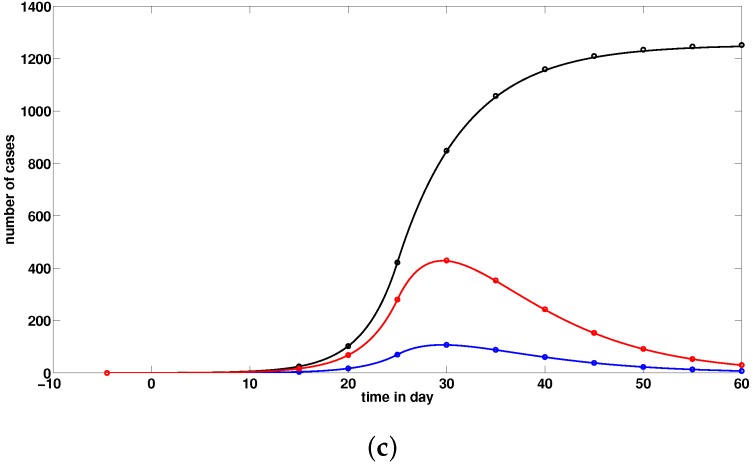
In this figure, we plot the graphs of t→CR(t) (black solid line), t→U(t) (blue solid line) and t→R(t) (red solid line). We use again f=0.8,η=1/7,ν=1/7, and $S_{0}=11.081\times 10^{6}$. In (**a**), we use $\chi_{1}=0.16$, $\chi_{2}=0.38$, $\chi_{3}=1.1$, $t_{0}=5.12$ for the parameter values for China which give $\tau =4.44\times 10^{-08}$ for $t\in [t_{0},25]$ and τ=0 for t>25, $I_{0}=3.62$, $U_{0}=0.2$. In (**b**), we use $\chi_{1}=0.23$, $\chi_{2}=0.34$, $\chi_{3}=0.1$ and $t_{0}=-2.45$, for the parameter values obtained from the data for Hubei province, which give $\tau =4.11\times 10^{-08}$ for $t\in [t_{0},25]$ and τ=0 for t>25, $I_{0}=0.3$, $U_{0}=0.02$. In (**c**), we use $\chi_{1}=0.36$, $\chi_{2}=0.28$, $\chi_{3}=0.1$, and $t_{0}=-4.52$ for the parameter values obtained from the data for Wuhan, which give $\tau =3.6\times 10^{-08}$ for $t\in [t_{0},25]$ and τ=0 for t>25, $I_{0}=0.25$, $U_{0}=0.02$. The cumulated number of reported cases goes up to 7000 in (**b**), 4000 in (**b**) and 1400 in (**c**), and the turning point is around 30 January in (**a**–**c**).

**Figure 5:**
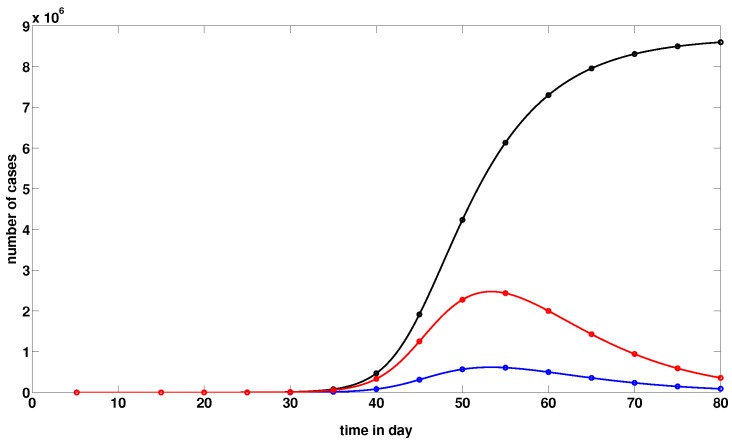
In this figure, we plot the graphs of t→CR(t) (black solid line), t→U(t) (blue solid line) and t→R(t) (red solid line). We use f=0.8,η=1/7,ν=1/7, and $S_{0}=11.081\times 10^{6}$. The remaining parameters are derived by using (6)–(8). We obtain $\tau =4.44\times 10^{-08}$, $I_{0}=3.62$ and $U_{0}=0.2$. The cumulated number of reported cases goes up to 8.5 million people and the turning point is day 54. Thus, the turning point is 23 February (i.e., 54–31).

The figure below takes into account the public health measures, such as isolation, quarantine, and public closings, which correspond to models (10) and (11). By comparison of Figure 4a with Figure 5, we note that these measures greatly mitigate the final size of the epidemic, and shift the turning point about 24 days before the turning point without these measures.

## 3. Discussion

An epidemic outbreak of a new human coronavirus COVID-19 has occurred in Wuhan, China. For this outbreak, the unreported cases and the disease transmission rate are not known. In order to recover these values from reported medical data, we present the mathematical model (1) for outbreak diseases. By knowledge of the cumulative reported symptomatic infectious cases, and assuming (1) the fraction *f* of asymptomatic infectious that become reported symptomatic infectious cases, (2) the average time 1/ν asymptomatic infectious are asymptomatic, and (3) the average time 1/η symptomatic infectious remain infectious, we estimate the epidemiological parameters in model (1). We then make numerical simulations of the model (1) to prodict forward in time the severity of the epidemic. We observe that public health measures, such as isolation, quarantine, and public closings, greatly reduce the final size of the epidemic, and make the turning point much earlier than without these measures. We observe that the predictive capability of model (1) requires valid estimates of the parameters *f*, ν, and η, which depend on the input of medical and biological epidemiologists. Our results can contribute to the prevention and control of the COVID-19 epidemic in Wuhan.

As a consequence of our study, we note that public health measures, such as isolation, quarantine, and public closings, greatly reduce the final size of this epidemic, and make the turning point much earlier than without these measures. With our method, we fix η, ν, and *f* and get the same turning point for the three data sets in Table 2, Table 3 and Table 4. We choose f=0.8, which means that around 80% of cases are reported in the model, since cases are very well documented in China. Thus, we only assume that a small fraction, 20%, were not reported. This assumption may be confirmed later on.

We also vary the parameters η, ν, and *f*, and we do not observe a strong variation of the turning point. Nevertheless, the number of reported cases are very sensitive to the data sets, as shown in the figures. Formula (4) for CR(t) is very descriptive until 26 January for the reported case data for China and Hubei but is not reasonable for Wuhan data. This suggests that the turning point is very robust, while the number of cases is very sensitive. We can find multiple values of η, ν, and *f* that provide a good fit for the data. This means that η, ν, and *f* should also be evaluated using other methods. The values 1/η=7 days and 1/ν=7 days are taken from information concerning earlier corona viruses, and are used now by medical authorities [2].

The predictive capability of models (1) and (10) requires valid estimates of the parameters *f* (fraction of asymptomatic infectious that become reported as symptomatic infectious), the parameter 1/ν (average time asymptomatic infectious are asymptomatic), and the parameter 1/η (average time symptomatic infectious remain infectious). In Figure 4, we graph $R_{0}$ as a function of *f* and 1/ν for the data in Table 2, to illustrate the importance of these values in the evolution of the epidemic. The accuracy of these values depend on the input of medical and biological epidemiologists.

**Figure 6:**
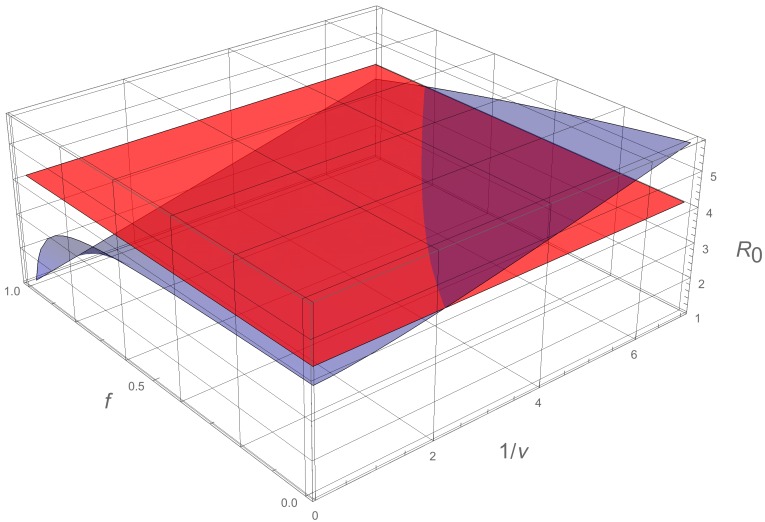
In this figure, we use 1/η=7 days, and we plot the basic reproductive number $R_{0}$ as a function of *f* and 1/ν using (9) with $\chi_{2}=0.38$, which corresponds to the data for China in Table 2. If both *f* and 1/ν are sufficiently small, $R_{0}<1$. The red plane is the value of $R_{0}=4.13$.

In influenza epidemics, the fraction *f* of reported cases may be significantly increased by public health reporting measures, with greater efforts to identify all current cases. Our model reveals the impact of an increase in this fraction *f* in the value of $R_{0}$, as evident in Figure 6 above, for the COVID-19 epidemic in Wuhan.

## 4. Materials and Methods

### 4.1. Method to Estimate the Parameters of (1) from the Number of Reported Cases

In the following the parameters f,ν and η are fixed.

**Step 1:** Since *f* and ν, we know that

$$\nu_{1}=f\nu \;\mathrm{and}\;\nu_{2}=\left(1-f\right)\;\nu .$$

**Step 2:** By using Equation (3), we obtain

(12) $$CR^{\prime }\left(t\right)=\nu_{1}I\left(t\right)\Leftrightarrow \chi_{1}\chi_{2}exp\left(\chi_{2}t\right)=\nu_{1}I\left(t\right)$$

and

$$\frac{exp\left(\chi_{2}t\right)}{exp\left(\chi_{2}t_{0}\right)}=\frac{I(t)}{I(t_{0})},$$

and therefore

(13) $$I\left(t\right)=I_{0}exp\left(\chi_{2}\left(t-t_{0}\right)\right).$$

Moreover, by using (12) at $t=t_{0}$,

(14) $$I_{0}=\frac{\chi_{1}\chi_{2}exp\left(\chi_{2}t_{0}\right)}{f\;\nu }=\frac{\chi_{3}\chi_{2}}{f\;\nu }.$$

**Step 3:** In order to evaluate the parameters of the model, we replace S(t) by $S_{0}=11.081\times 10^{6}$ on the right-hand side of (1) (which is equivalent to neglecting the variation of susceptibles due to the epidemic, which is consistent with the fact that t→CR(t) grows exponentially). Therefore, it remains to estimate τ and η in the following system:

(15) $$\left\{\begin{array}{c} I^{\prime }\left(t\right)=\tau S_{0}\left[I\left(t\right)+U\left(t\right)\right]-\nu I\left(t\right)\\ U^{\prime }\left(t\right)=\nu_{2}I\left(t\right)-\eta U\left(t\right). \end{array}\right.$$

By using the first equation, we obtain

$$U\left(t\right)=\frac{1}{\tau S_{0}}\left[I^{\prime }\left(t\right)+\nu I\left(t\right)\right]-I\left(t\right),$$

and therefore, by using (13), we must have

$$I\left(t\right)=I_{0}\exp\left(\chi_{2}\left(t-t_{0}\right)\right)\;\mathrm{and}\;U\left(t\right)=U_{0}\exp\left(\chi_{2}\left(t-t_{0}\right)\right),$$

so, by substituting these expressions into (15), we obtain

(16) $$\left\{\begin{array}{c} \chi_{2}I_{0}=\tau S_{0}\left[I_{0}+U_{0}\right]-\nu I_{0}\\ \chi_{2}U_{0}=\nu_{2}I_{0}-\eta U_{0}. \end{array}\right.$$

**Remark** **3.** *Here, we fix τ in such a way that the value $\chi_{2}$ becomes the dominant eigenvalue of the linearized Equation (21) and $\left(I_{0},U_{0}\right)$ is the positve eigenvector associated with this dominant eigenvalue $\chi_{2}$. Thus, we apply implicitly the Perron–Frobenius theorem. Moreover, the exponentially growing solution (I(t),U(t)) that we consider (which is starting very close to (0,0)) follows the direction of the positive eigenvector associated with the dominant eigenvalue $\chi_{2}$.*

By dividing the first equation of (16) by $I_{0}$ we obtain

$$\chi_{2}=\tau S_{0}\left[1+\frac{U_{0}}{I_{0}}\right]-\nu$$

and hence

(17) $$\frac{U_{0}}{I_{0}}=\frac{\chi_{2}+\nu }{\tau S_{0}}-1.$$

By using the second equation of (16), we obtain

(18) $$\frac{U_{0}}{I_{0}}=\frac{\nu_{2}}{\eta +\chi_{2}}.$$

By using (17) and (18), we obtain

(19) $$\tau =\frac{\chi_{2}+\nu }{S_{0}}\frac{\eta +\chi_{2}}{\nu_{2}+\eta +\chi_{2}}.$$

By using (18), we can compute

(20) $$U_{0}=\frac{\nu_{2}}{\eta +\chi_{2}}I_{0}=\frac{(1-f)\nu }{\eta +\chi_{2}}I_{0}.$$

### 4.2. Computation of the Basic Reproductive Number $R_{0}$

In this section, we apply results in Diekmann, Heesterbeek, and Metz [7] and Van den Driessche and Watmough [8]. The linearized equation of the infectious part of the system is given by

(21) $$\left\{\begin{array}{c} I^{\prime }\left(t\right)=\tau S_{0}\left[I\left(t\right)+U\left(t\right)\right]-\nu I\left(t\right),\\ U^{\prime }\left(t\right)=\nu_{2}I\left(t\right)-\eta U\left(t\right). \end{array}\right.$$

The corresponding matrix is

$$A=\left[\begin{array}{cc} \tau S_{0}-\nu & \tau S_{0}\\ \nu_{2} & -\eta \end{array}\right]$$

and the matrix *A* can be rewritten as

A=V−S

where

$$V=\left[\begin{array}{cc} \tau S_{0} & \tau S_{0}\\ \nu_{2} & 0 \end{array}\right]\;\mathrm{and}\;S=\left[\begin{array}{cc} \nu & 0\\ 0 & \eta \end{array}\right].$$

Therefore, the next generation matrix is

$$VS^{-1}=\left[\begin{array}{cc} \frac{\tau S_{0}}{\nu } & \frac{\tau S_{0}}{\eta }\\ \frac{\nu_{2}}{\nu } & 0 \end{array}\right]$$

which is a Leslie matrix, and the basic reproductive number becomes

(22) $$R_{0}=\frac{\tau S_{0}}{\nu }\left(1+\frac{\nu_{2}}{\eta }\right).$$

By using (19), we obtain

$$R_{0}=\frac{\chi_{2}+\nu }{S_{0}}\frac{\eta +\chi_{2}}{\nu_{2}+\eta +\chi_{2}}\frac{S_{0}}{\nu }\left(1+\frac{\nu_{2}}{\eta }\right)$$

and, by using $\nu_{2}=\left(1-f\right)\;\nu$, we obtain

(23) $$R_{0}=\frac{\chi_{2}+\nu }{\nu }\frac{\eta +\chi_{2}}{\left(1-f\right)\;\nu +\eta +\chi_{2}}\left(1+\frac{(1-f)\;\nu }{\eta }\right).$$

## Figures and Tables

**Table 1:** Parameters of the model.

Symbol	Interpretation	Method
$t_{0}$	Time at which the epidemic started	fitted
$S_{0}$	Number of susceptible at time $t_{0}$	fixed
$I_{0}$	Number of asymptomatic infectious at time $t_{0}$	fitted
$U_{0}$	Number of unreported symptomatic infectious at time $t_{0}$	fitted
τ	Transmission rate	fitted	
1/ν	Average time during which asymptomatic infectious are asymptomatic	fixed
*f*	Fraction of asymptomatic infectious that become reported symptomatic infectious	fixed
$\nu_{1}=f\;\nu$	Rate at which asymptomatic infectious become reported symptomatic	fitted
$\nu_{2}=\left(1-f\right)\;\nu$	Rate at which asymptomatic infectious become unreported symptomatic	fitted
1/η	Average time symptomatic infectious have symptoms	fixed

**Table 2:** Reported case data 20–29 January 2020, reported for mainland China by the Chinese CDC [1].

Date January	20	21	22	23	24	25	26	27	28	29
Confirmed cases (cumulated) for mainland China	291	440	571	830	1287	1975	2744	4515	5974	7711
Mortality cases (cumulated) for mainland China		9	17	25	41	56	80	106	132	170

**Table 3:** Reported case data 23–31 January 2020, reported for Hubei Province by the Wuhan Municipal Health Commission [6].

Date January	23	24	25	26	27	28	29	30	31
Confirmed cases (cumulated) for Hubei	549	729	1052	1423	2714	3554	4586	5806	7153
Mortality cases (cumulated) for Hubei	24	39	52	76	100	125	162	204	249

**Table 4:** Reported case data 23–31 January 2020, reported for Wuhan Municipality by the Wuhan Municipal Health Commission [6].

Date January	23	24	25	26	27	28	29	30	31
Confirmed cases (cumulated) for Wuhan	495	572	618	698	1590	1905	2261	2639	3215
Mortality cases (cumulated) for Wuhan	23	38	45	63	85	104	129	159	192

**Table 5:** Estimation of the parameters $\chi_{1}$, $\chi_{2}$, $\chi_{3}$, and $t_{0}$ by using the cumulated reported cases in Table 2, Table 3 and Table 4.

Name of the Parameter	$\chi_{1}$	$\chi_{2}$	$\chi_{3}$	$t_{0}$
From Table 2 for China	0.16	0.38	1.1	5.12
From Table 3 for Hubei	0.23	0.34	0.1	−2.45
From Table 4 for Wuhan	0.36	0.28	0.1	−4.52
